# Sulforaphane improves chemotherapy efficacy by targeting cancer stem cell-like properties via the miR-124/IL-6R/STAT3 axis

**DOI:** 10.1038/srep36796

**Published:** 2016-11-08

**Authors:** Xingxing Wang, Yuan Li, Yi Dai, Qinqiang Liu, Shilong Ning, Jiao Liu, Zhaoxia Shen, Dongmei Zhu, Fei Jiang, Jianping Zhang, Zhong Li

**Affiliations:** 1 Key Laboratory of Modern Toxicology, Ministry of Education, Department of Nutrition and Food Hygiene, School of Public Health, Nanjing Medical University, Nanjing, 211166, China; 2 Department of surgery, the second affiliated hospital, Nanjing medical university, Nanjing, 211166, China

## Abstract

Gastric carcinoma (GC) is the second leading cause of cancer-related mortality worldwide. The efficacy of standard chemotherapy for GC, such as cisplatin (CDDP), is dissatisfactory partly due to the toxic/side-effects. Sulforaphane (SFN), which exhibits effective anti-cancer functions, is a phytochemical converted from cruciferous plants. Our present study aimed to identify whether SFN could enhance the anti-cancer effects of low-dose CDDP and to determine the underlying mechanisms. Herein, co-exposure of SFN and CDDP significantly inhibited the viabilities of gastric cancer cells. For the molecular mechanisms, CDDP alone increased the cancer stem cell (CSC)-like properties in gastric cancer cells via activating the interleukin-6 (IL-6)/IL-6 receptor (IL-6R)/signal transducer and activator of transcription 3 (STAT3) signaling. However, SFN could activate the microRNA-124 (miR-124), which directly targets the 3′-untranslated regions (UTR) of the IL-6R and STAT3. Moreover, knockdown of miR-124 eliminated the effects of SFN on CSC-like properties in GC cells, and in turn enhanced the anti-cancer effects of low-dose CDDP. These findings not only suggested a mechanism whereby SFN enhanced the anti-cancer functions of CDDP, but also helped to regard SFN as a potential chemotherapeutic factor in gastric cancer.

Gastric carcinoma (GC) is one of the most common malignances worldwide, ranking second in causes of cancer-related mortality worldwide^1^,^2^. The overall 5-year survival rate of GC is only 20% and it has a 50–90% risk of recurrence and death even after resection operation^3^,^4^. In spite of surgery, chemotherapy still plays a pivotal role in improving overall survival of gastric cancer patients especially of those with advanced GC^5^. Cisplatin (CDDP), a DNA-targeting cytotoxic platinum compound, is one of the first-line chemotherapeutic agents for GC^6^. It works by the formation of DNA adducts, leading to apoptosis and cellular senescence^7^. However, the efficacy of current standard chemotherapy including CDDP is restricted partly due to the severe toxic/side-effects. The toxic effects of CDDP are dose-dependent, including renal, otologic, bone marrow suppression, and neurotoxicity^8^,^9^,^10^. Since high levels of CDDP are cytotoxic to both carcinoma and normal cells, the reducing of the dosage of CDDP and achieving the satisfactory chemotherapy efficacy are urgently needed.

Many of the naturally occurring phytochemicals are well-established to be promising candidates for anticancer drug development. Sulforaphane (SFN) is a phytochemical converted from cruciferous plants, such as broccoli sprouts, kale, and carrots^11^. Due to its extensive sources, hypotoxicity, and diverse biological functions, SFN has been intensively investigated in many cancers. For example, SFN inhibits the phase I enzymes but induces the phase II enzymes, promotes the apoptosis and cell cycle arrest, and inhibits the metastasis and angiogenesis^12^. In addition, SFN has been demonstrated to target multiple pathways involved in cancer cells in combination with other anticancer compounds. For example, SFN potentiates the efficacy of imatinib and sorafenib against chronic myeloid leukemia cells and pancreatic cancer cells, respectively^13^,^14^; in addition, SFN also acts synergistically with human tumor necrosis factor-related apoptosis ligand in advanced prostate cancer cells^15^. However, the combined effects of SFN and CDDP in GC cells remain obscure. Therefore, our present study was designed to investigate whether SFN could be the potent agent, which facilitated the chemotherapy efficacy of low-dose CDDP in GC cells and to determine by whereby these effects occurred.

## Results

### SFN synergized with CDDP in GC cells

**Figure 1:**
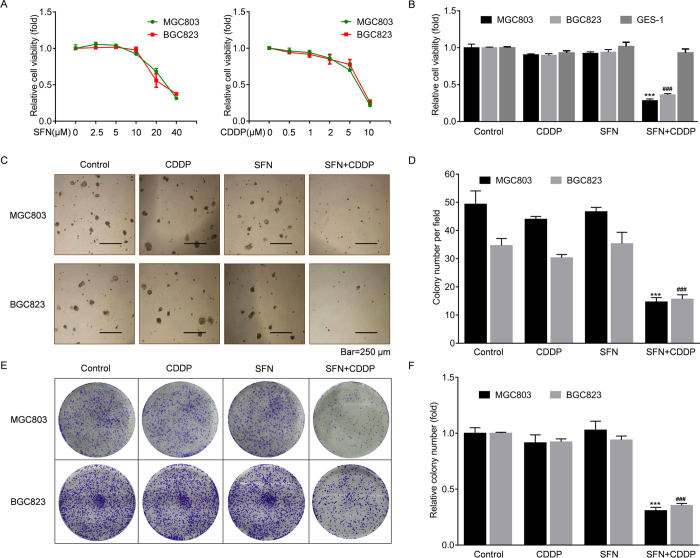
SFN synergized with CDDP in GC cells. (**A**) MGC803 and BGC823 cells were treated with 0, 2.5, 5, 10, 20, or 40 μM of SFN and 0, 0.5, 1, 2, 5, or 10 μM of CDDP for 72 h, respectively. The cell viabilities were evaluated in quadruplicate by WST-8 hydrolysis using a CCK-8 assay. (**B**) MGC803, BGC823 and GES-1 cells were treated with 2 μM of CDDP, 10 μM of SFN, or their combination for 72 h, and cell viabilities were evaluated by the CCK-8 assay. (**C**–**F**) Colony formation assay: After treatment with 2 μM of CDDP, 10 μM of SFN, or their combination for 72 h, 1 × 10^3^ cells of each group were seeded into six-well plates with soft agar (**C**) or fresh culture medium (**D**) for 2 weeks. Colonies were then photographed under a microscope or camera. (**E**) Colony numbers per field in soft agar (mean ± SD, n = 5; five randomly chosen fields). (**F**) Relative colony number in plates (mean ± SD, n = 3). ^***^p < 0.001 compared with medium control MGC803 cells, ^###^*p *< 0.001 compared with medium control BGC823 cells.

First, we treated human GC cell lines, MGC803 and BGC823, by different concentrations of SFN or CDDP. As shown in Fig. 1A, the viabilities of these cells were not appreciably affected within 10 μM of SFN or 2 μM of CDDP respectively. Next, we used 10 μM of SFN and/or 2 μM of CDDP to treat MGC803, BGC823, and human gastric epithelial cell line, GES-1, respectively. As shown in Fig. 1B, SFN synergistically acted with CDDP to dramatically inhibit the viabilities of GC cells compared to single treatment, however, there was no detectable effect of SFN and CDDP combination on GES-1 cells. Then, we further evaluated the long-term inhibitory effects of SFN and CDDP combination on the colony formation. Interestingly, single drug usage did not limit the colony growth, however, combined treatment exhibited a significant reduction in both soft agar (Fig. 1C,D) and plate (Fig. 1E,F) colony numbers. According to these results, we proposed two questions: (1) what happened while using the low-dose of CDDP and SFN, and (2) whereby these synergistic effects occurred.

### SFN repressed the CDDP-induced CSC-like properties in GC cells

**Figure 2:**
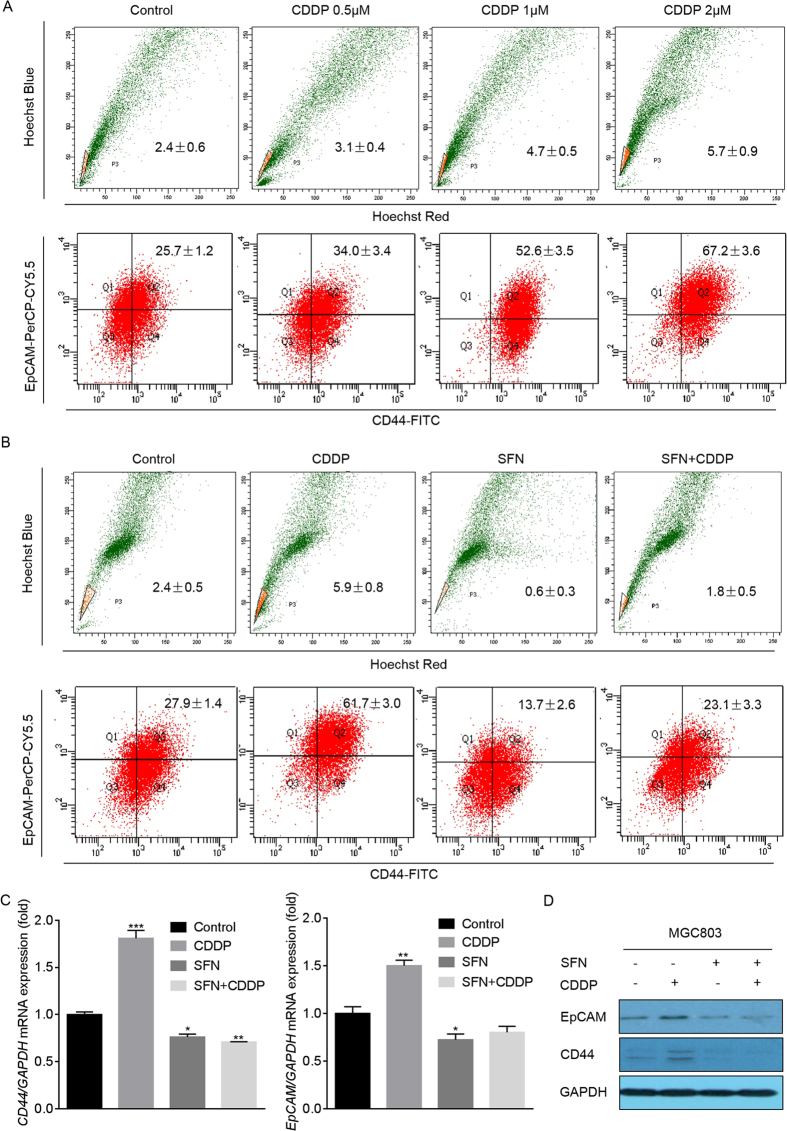
SFN repressed the CDDP-induced CSC-like properties in GC cells. (**A**) MGC803 cells were treated with 0, 0.5, 1, or 2 μM of CDDP for 72 h. (**B**–**D**) MGC803 cells were treated with 2 μM of CDDP, 10 μM of SFN, or their combination for 72 h. (**A**,**B**) Flow cytometry analyses of the percentage of SP (upper row) and CD44^+^/EpCAM^+^ (lower row) cells in MGC803 cell populations (mean ± SD, n = 3). (**C**) qRT-PCR analyses of *CD44* and *EpCAM* (mean ± SD, n = 3). ^*^p < 0.05, ^**^p < 0.01, and ^***^p < 0.001 compared with medium control MGC803 cells. (**D**) Western blot analysis of CD44 and EpCAM.

It is well acknowledged that undesirable chemotherapy efficacy is related to a subpopulation in cancer cells named CSCs, which have enhanced self-renewal, multi-differentiation, and tumorigenicity properties^16^. There are mainly three methods for the identification of CSCs or CSC-like properties: (1) use of CSCs surface markers, such as CD44^+^CD24^−^, CD133, CD44^+^/EpCAM^+^, and CD90^17^,^18^; (2) identifying the side population (SP) in cancer cells, which enriches CSC-like properties; and (3) determining the growth properties of cells in serum-free suspension culture^19^. Here, in GC cells, we validated that, CDDP elevated the ratios of SP and CD44^+^/EpCAM^+^ cells in a dose-dependent manner (Fig. 2A), however, the ratios were significantly reversed in the presence of SFN (Fig. 2B). In addition, SFN was also suppressed the CDDP-induced increased expressions of CD44 and EpCAM mRNA and proteins (Fig. 2C,D and Fig. S1). Collectively, these results suggested that CDDP expanded the CSC-like properties in GC cells, however, SFN effectively blocked this effect.

### SFN suppressed the CDDP-activated IL-6/STAT3 signaling in GC cells

**Figure 3:**
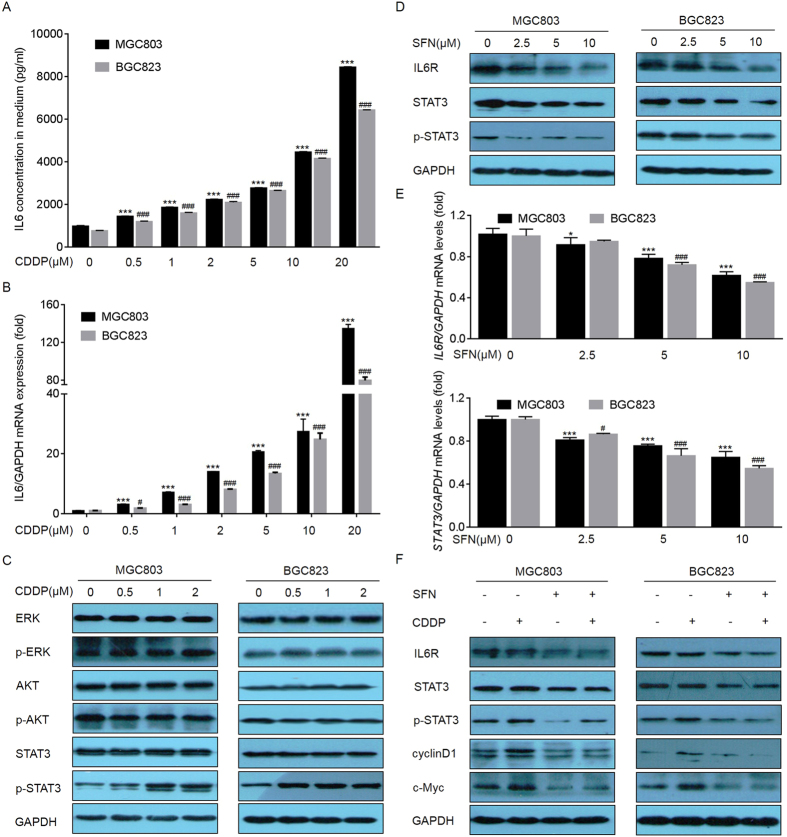
SFN suppressed the CDDP-activated IL-6/STAT3 signaling. (**A,B**) MGC803 and BGC823 cells were treated with 0, 0.5, 1, 2, 5, 10 or 20 μM of CDDP for 72 h. (**A**) ELISA analyses of secretion levels of IL-6. (**B**) qRT-PCR analyses of expression of *IL6* (mean ± SD, n = 3). **(C)** MGC803 and BGC823 cells were treated with 0, 0.5, 1, or 2 μM of CDDP for 72 h. Western blot analysis of ERK, p-ERK, Akt, p-Akt, STAT3, and p-STAT3. (**D**,**E**) MGC803 and BGC823 cells were treated with 0, 2.5, 5, or 10 μM of SFN for 72 h. (**D**) Western blot analysis of IL-6R, STAT3, and p-STAT3. (**E**) qRT-PCR analyses of expressions of *IL6R* and *STAT3* (mean ± SD, n = 3). (**F**) Western blot analysis was conducted after MGC803 and BGC823 cells were treated with 2 μM of CDDP, 10 μM of SFN, or their combination for 72 h. ^*^p < 0.05 and ^***^p < 0.001 compared with medium control MGC803 cells. ^#^*p* < 0.05 and ^###^*p* < 0.001 compared with medium control BGC823 cells.

IL-6 is a multifunctional cytokine which initiates its biological functions in GC by binding to its receptor and then activating the downstream signals, including mitogen-activated protein kinase (MAPK), phosphatidylinositol 3-kinase (PI3K), and STAT3^20^,^21^,^22^,^23^. Here, CDDP dramatically increased the expression/secretion of IL-6 in a dose-dependent manner (Fig. 3A,B). Interestingly, without significant changes of phosphorylations of ERK and Akt, the phosphorylation of STAT3 was markedly increased in CDDP-treated GC cells (Fig. 3C). Then, we further determined the effects of SFN on this phenomenon. Here, SFN did not change the CDDP-induced expression of IL-6 (data not shown) but inhibited the expressions of IL-6R, STAT3, and p-STAT3 in a dose-dependent manner (Fig. 3D,E). Moreover, SFN reversed the CDDP-induced increased expressions of p-STAT3 and its downstream targets, cyclinD1 and c-Myc (Fig. 3F). Based on these results, we hypothesized that (1) the selective activation of IL-6/STAT3 signal pathway by might be involved in the CDDP-induced expansion of CSC-like properties in gastric cancer cells; and that (2) the inhibition of CDDP-induced activation of IL-6/STAT3 signaling was involved in the SFN-mediated synergistic effects in GC cells.

### IL-6/STAT3 was responsible for CDDP-induced CSC-like properties in GC cells

**Figure 4:**
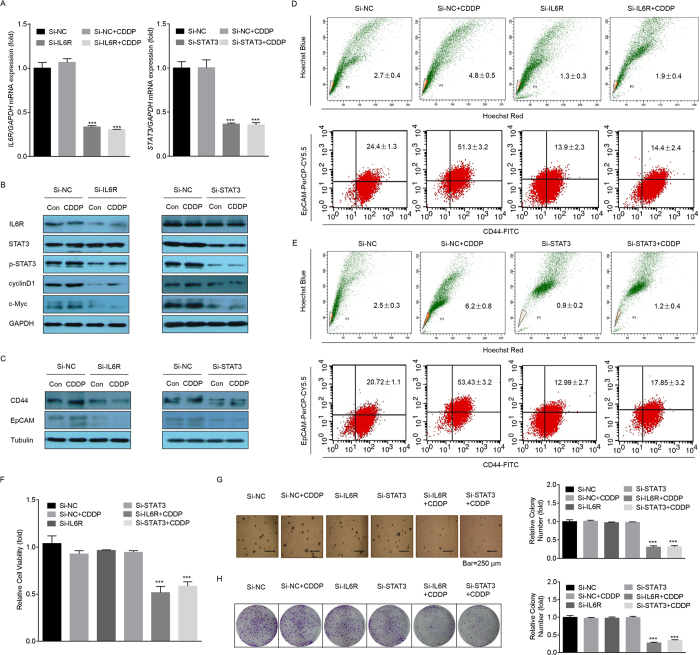
IL-6/STAT3 was responsible for CDDP-induced CSC-like properties. (**A**–**H**) MGC803 cells were transfected with con-siRNA, IL6R-siRNA, or STAT3-siRNA for 12 h and then 2 μM of CDDP was added for 72 h. (**A**) qRT-PCR analyses of expressions of *IL6R* or *STAT3* (mean ± SD, n = 3). ^***^p < 0.001 compared with medium control MGC803 cells. (**B**,**C**) Western blot analysis analysis of related proteins. (**D**,**E**) FACS analyses of the percentage of SP (upper row) and CD44^+^/EpCAM^+^ (lower row) cells in MGC803 cell populations (mean ± SD, n = 3). (**F**) Cell viability was evaluated by the CCK-8 assay. (**G**, left) Soft agar colonies under a microscope after 2 weeks of cultivation. (**G**, right) Relative colony numbers in soft agar (mean ± SD, n = 5). **(H**, left) Plate colonies after 2 weeks. (**H**, right) Relative colony numbers in plates (mean ± SD, n = 3). ^***^p < 0.001 compared with MGC803 cells transfected by con-siRNA.

To validate the hypothesis (1) above, we blocked IL-6/STAT3 signaling by the transfection of small interfering RNAs (siRNAs) targeting IL-6R or STAT3 in GC cells. Here, knockdown of IL-6R only affected the expression of p-STAT3, but not total STAT3 (Fig. 4A,B). In addition, knockdown of either IL-6R or STAT3 suppressed the expressions of cyclinD1 and c-Myc, and reversed the CDDP-induced elevations of CD44 and EpCAM expression (Fig. 4C and Fig. S2), SP and CD44^+^/EpCAM^+^ cell ratios ((Fig. 4D,E). Furthermore, knockdown of either IL-6R or STAT3 enhanced the anti-cancer effects of CDDP, as determined by inhibiting the cell viability (Fig. 4F) and reducing the anchorage-independent growth in soft agar (Fig. 4G,H, and Fig. S3).

### SFN suppressed the IL-6R and STAT3 through microRNA-124 (miR-124)

**Figure 5:**
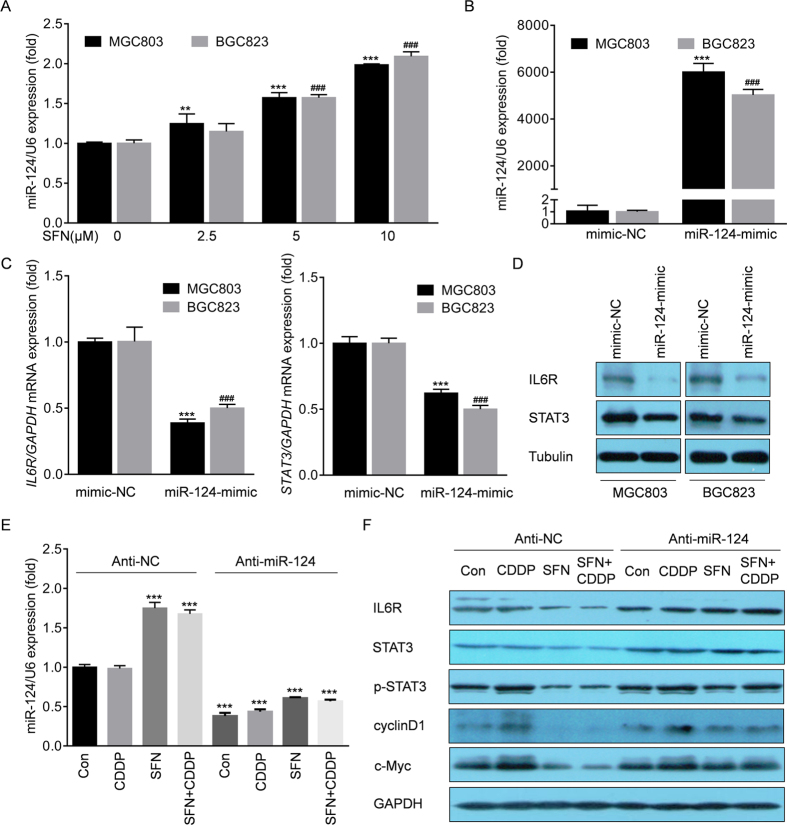
SFN suppressed IL-6R and STAT3 through activation of miR-124. (**A**) MGC803 and BGC823 cells were treated with 0, 2.5, 5, or 10 μM of SFN for 72 h. The qRT-PCR analyses of miR-124 (mean ± SD, n = 3). ^**^p < 0.01 and ^***^p < 0.001 compared with medium control MGC803 cells. ^###^*p *< 0.001 compared with medium control BGC823 cells. (**B**–**D**) MGC803 and BGC823 cells were transfected with con-mimic or miR-124-mimic for 12 h. (**B**,**C**) The qRT-PCR analyses of miR-124, *IL6R*, and *STAT3* (mean ± SD, n = 3). ^***^p < 0.001 compared with MGC803 cells transfected with con-mimic. ^###^*p* < 0.001 compared with BGC823 cells transfected with con-mimic. (**D**) Western blot analysis of IL-6R, STAT3, and Tubulin. (**E**,**F**) MGC803 cells were transfected with anti-con or anti-miR-124 for 12 h and treated with 2 μM of CDDP, 10 μM of SFN, or their combination for 72 h. (**E**) qRT-PCR analyses of miR-124 (mean ± SD, n = 3). ^***^p < 0.001 compared with MGC803 cells transfected with anti-con. (**F**) Western blot analysis of IL-6R, STAT3, p-STAT3, cyclin D, and c-Myc.

Next, we validated the hypothesis (2) above, and further determined the potential mechanisms. Here, SFN inhibited the mRNA level of IL-6R (Fig. 3), indicating a transcriptional inhibition or an epigenetic silencing, such as DNA hypermethylation, histone de-acetylation, as well as miRNAs might be involved. Previous studies showed that SFN could inhibit the histone de-acetylation and promote the global de-methylation in various cancers^24^,^25^,^26^. However, the effects of SFN on miRNAs in cancer cells are less reported. By consulting literature materials and analyzing with TargetScan (http://www.targetscan.org/), several miRNAs were screened out to be involved in directly targeting IL-6R, including miR-34a, miR-449a, miR-125b and miR-124 27,28,29,30. Here, SFN had no remarkable increased effect on miR-34a, miR-449a and miR-125b (Fig. S4), but caused a significant elevation of miR-124 in a dose-dependent manner (Fig. 5A). When transfected by miR-124-mimic (Fig. 5B), the expression of IL-6R was significantly suppressed (Fig. 5C,D), which was consistent with previous reports^30^,^31^. In addition, miR-124 was also found to directly target STAT3^30^. So we then determined the effects of miR-124 on the function of SFN in the presence or absence of CDDP. As shown in Fig. 5E,F, and Fig. S4, knockdown of miR-124 eliminated the SFN-induced blockage of the IL-6R/STAT3 pathway. Collectively, these results suggested that SFN suppresses the IL-6R and STAT3 expression via miR-124.

### SFN decreased the CDDP-induced CSC-like properties via miR-124

**Figure 6:**
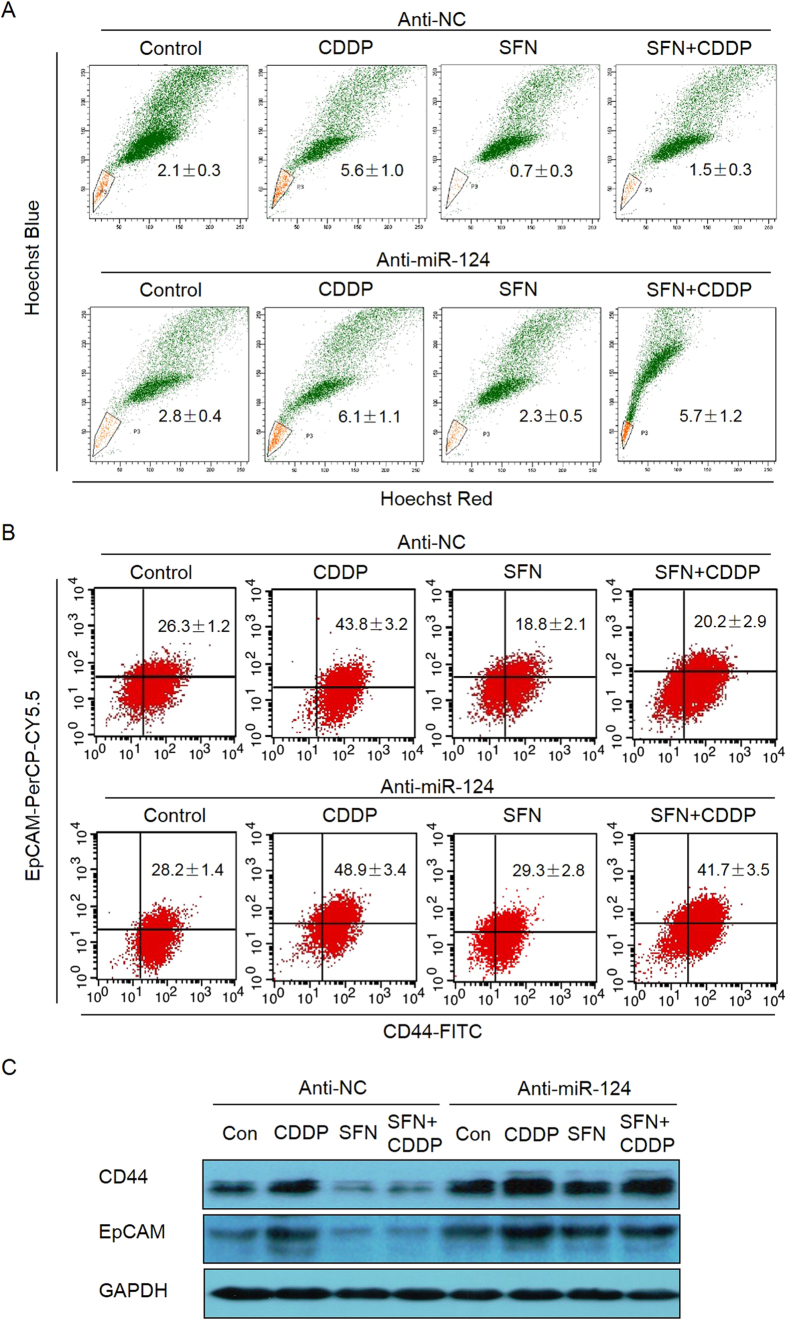
SFN decreased the CDDP-induced CSC-like properties via miR-124. (**A**–**C**) MGC803 cells were transfected with anti-con or anti-miR-124 for 12 h and treated with 2 μM of CDDP, 10 μM of SFN, or their combination for 72 h. FACS analyses of the percentage of SP (**A**) and CD44^+^/EpCAM^+^ (**B**) cells in MGC803 cell populations (mean ± SD, n = 3). (**C**) Western blot analysis of CD44 and EpCAM.

Finally, we investigated the role of miR-124 in SFN-suppressed CSC-like properties in GC cells in the presence of absence of CDDP. As shown in Fig. 6A,B knockdown of miR-124 eliminated the effects of SFN on CSC-like properties, manifested as rebounds of SP and CD44^+^/EpCAM^+^ cell ratios. Also, the protein levels of CD44 and EpCAM were reversed after down-regulation of miR-124 (Fig. 6C and Fig. S5). Collectively, these results indicate that SFN decreases the CDDP-induced CSC-like properties in a miR-124-dependent manner.

## Discussion

Accumulating evidence suggested that chemotherapy failure might be blamed for the existence of CSCs or cancer cells with CSC-like properties, which have been identified in various types of cancer cells. Tumor cells have heterogeneity and the less-differentiated CSCs are recognized as the origin of tumorigenesis, metastasis and recurrence in numerous human cancers^16^,^32^. Gastric CSCs have been isolated in several cell lines as well as in primary tumors^19^,^33^. Our results showed that CDDP elevated the ratios of SP and CD44^+^/EpCAM^+^ cells in a dose-dependent manner. Since low-dose CDDP alone did not have effect on GC cell viability, these results indicated that low-dose CDDP promoted the de-differentiation of GC bulk cells and expanded the proportion of CSCs. Though rarely reported in GC cells, CDDP-induced acquisition of CSC-like properties has been detected in other tumors, including lung, head and neck, and osteosarcoma^34^,^35^,^36^,^37^. In our present study, we did not use a lethal dose administration of chemotherapeutics, which eliminate the tumor bulk by killing differentiated tumor cells but spare the less differentiated CSCs that are resistant to conventional chemotherapy. Indeed, we putted an emphasis on the CSC-like properties generated in mature cancer cells after low dose CDDP. Furthermore, we also emphasizes the role of autocrine IL-6 by CDDP in gastric cancer cells, since increased serum levels of IL-6 are predictive of poor prognosis in gastric cancer^38^,^39^.

SFN was reported to target CSCs through direct or indirect mechanisms, alone or in combination with other anticancer compounds^40^,^41^. For example, SFN regulates the self-renewal ability of pancreatic CSCs through modulating the sonic hedgehog-GLI and/or Wnt/β-catenin pathways^42^,^43^. However, whether SFN could target gastric CSCs or the de-differentiated GC cells with CSC-like properties induced by CDDP remains unclear. Here, SFN inhibited the IL-6R/STAT3 pathway but not affected the viability in GC cells; in addition, combined with SFN, the attenuation of CSC-like properties facilitated CDDP to exert the therapeutic functions.

MiRNAs are small non-coding RNAs that regulate gene expression by binding to the 3′-UTR regions of target mRNAs to induce degradation or translation inhibition^44^. As a tumor suppressor, aberrant low expression of miR-124 is often detected in many tumors, such as gynecological, cerebral, and gastric tumors^45^,^46^,^47^. MiR-124 was reported to perform crucial anti-cancer functions in GC^48^,^49^. It also plays important roles in mediating CSCs. For example, overexpression of miR-124 reduced neurosphere formation, CD133^+^ cell subpopulations, and stem cell markers such as BMI1, Nanog, and nestin in glioma cells^50^. However, the relationship between miR-124 and CSCs in GC cells has not been reported. Here, SFN increased the expression of miR-124 in GC cells. Knockdown of miR-124 attenuated the function of SFN on CDDP, suggesting the activation of miR-124 was required for SFN to target CSC-like properties.

In conclusion, our findings suggested the possibility of developing CSCs-targeting agents from a natural phytochemicals, SFN. In addition, our data also suggested a role for the miR-124/IL-6R/STAT3 pathway in modulating the chemotherapeutic effects in GC cells.

## Methods

### Cell culture and reagents

Human GC cell lines MGC803 and BGC823 were obtained from the Shanghai Cell Bank of the Chinese Academy of Sciences (Shanghai, China). The cells were cultivated in RPMI-1640 media supplemented with 10% fetal bovine serum (FBS; Gibco-BRL, Capital, IN, USA) and 1% penicillin and streptomycin (Gibco-BRL) at 37 °C in a humidified incubator with 5% CO_2_. SFN (purity ≥ 95%) and CDDP (purity ≥ 99.9%) were purchased from Sigma–Aldrich Chemical Co. (St. Louis, MO, USA). All other reagents used were of analytical grade or the highest grade available.

### Cell viability and colony formation assay

For cell viability assays, MGC803 or BGC823 cells were seeded into 96-well plates at a density of 2 × 10^3^ cells per well for 24 h. Then, cells were treated with different concentrations of SFN or CDDP, alone or in combination, for 72 h, repeated four times for each concentration. Cell viability was detected using the Cell Counting Kit-8 (CCK-8, Dojindo Molecular Technologies, Inc., Kumamoto, Japan) according to the manufacturer’s instructions. To determine the long-term effects of SFN or CDDP on cell proliferation, colony formation assays were conducted, including soft agar and plate colony assays. Soft agar plates were prepared in six-well plates with under-layers of 0.70% agarose in RPMI-1640 medium (Gibco) supplemented with 10% FBS. Cells after treatments were plated in triplicate at a density of 1 × 10^3^ cells in 2 mL of 0.35% agarose over the agar base. Colonies were photographed and counted under a microscope (Olympus, Tokyo, Japan) after 2 weeks. For plate colony assays, cells after treatments were seeded into six-well plates at a density of 1 × 10^3^ cells per well, with fresh culture medium. After 2 weeks, colonies were fixed with 4% paraformaldehyde and stained with crystal violet solution. The colonies were then photographed and counted under a microscope (Olympus).

### Flow cytometry analysis

For SP analyses, cells after treatment were digested with trypsin, washed with phosphate-buffered saline, and then resuspended in Dulbecco’s modified Eagle’s medium/F-12 medium (Gibco-BRL) containing 2% FBS at a density of 1 × 10^6^ cells/mL, and stained with 5 μg/mL Hoechst 33342 (Sigma-Aldrich) in the presence or absence of 50 μM verapamil (Sigma-Aldrich) at 37 °C for 90 min. At the end of the incubation period, cells were counterstained with 2 μg/mL propidium iodide (Sigma-Aldrich). Analyses were performed using a FACS AriaIII system (BD Biosciences, San Jose, CA, USA). For CD44^+^/EpCAM^+^ cell analyses, cells after treatment were washed, resuspended, and then incubated at 4 °C in the dark for 40 min with fluorescence-conjugated monoclonal antibodies obtained from BD Biosciences against human CD44-FITC and EpCAM-Percp-Cy5.5, and their isotype IgG_1_ at concentrations recommended by the manufacturer. The samples were analyzed on a FACS Aria III (BD Biosciences). The results were analyzed using FlowJo software (Ashland, OR, USA). All FACS plots in our results have their corresponding negative controls. We have listed the complete FACS plots in supplementary materials.

### Enzyme-linked immunosorbent assay (ELISA)

To analyze IL-6 secretion, cells were treated with 0.0, 0.5, 1, or 2 μM of CDDP for 72 h. Then, the supernatants were collected and ELISA was performed using the human IL-6 Quantikine kit (R&D Systems, Minneapolis, MN, USA) according to the manufacturer’s protocol. Recombinant human IL-6 was used for calibration. The absorbance at 450/570 nm was measured with a multimode microplate reader (Tecan, San Jose, CA, USA).

### RNA interference and microRNA transfection

The siRNAs targeting IL-6R or STAT3 were purchased from RiBoBio Co (Guangzhou, China) and transfected into MGC803 cells using the Lipofectamine^®^ 2000 reagent (Invitrogen, Carlsbad, CA, USA) according to the manufacturer’s protocol. Con-mimic, miR-124-mimic, anti-con, and anti-miR-124 were also synthesized by RiBoBio. Briefly, cells were seeded into six-well plates at a density of 1 × 10^5^ cells per well for 24 h and then were transfected with 50 nM anti-miR-124 or 20 nM miR-124-mimic for 12 h. After transfections, cells were cultured in fresh RPMI-1640 medium supplemented with 10% FBS (Gibco-BRL) for another 24 h before using for other experiments.

### Western blots

Cells lysates were scraped off and centrifuged at 14000 g for 15 min. Protein concentrations were measured using the BCA protein assay (Beyotime, Shanghai, China). Protein samples were diluted to equal concentrations (20 μg), boiled for 10 min, and separated by 10% sodium dodecyl sulfate-polyacrylamide gel electrophoresis and transferred to polyvinylidene fluoride membranes (Millipore, Billerica, MA, USA). Antibodies used were against: IL-6R (Santa Cruz Biotechnology, Santa Cruz, CA, USA), STAT-3, p-STAT-3 (Tyr 705), AKT, p-AKT (Ser 473), ERK, p-ERK, cyclinD1, c-Myc, CD44, and EpCAM (Cell Signaling Technology, Boston, MA, USA), GAPDH and Tubulin (Beyotime). All antibody dilutions were 1:1000. The immune complexes were detected by enhanced chemiluminescence (Cell Signaling Technology).

### Real-time PCR analysis

The primers used are listed in Table 1. For the detection of mRNAs, total RNA (2 μg) was transcribed into cDNA using AMV reverse transcriptase (Promega, Madison, WI, USA). For the detection of miRNAs, 2 μg of total RNA, miRNAs-specific stem-loop RT primers, and MMLV reverse transcriptase (Promega, Sunnyvale, CA, USA) were used in reverse transcription following the manufacturer’s protocol. The qRT-PCR was performed using an ABI 7300 real-time PCR detection system (Applied Biosystems by Life Technologies, Grand Island, NY, USA). Fold changes in the expression of each gene were calculated by a comparative threshold cycle (Ct) method.

### Statistical analysis

Data were presented as the means ± standard deviation (SD). A Student’s *t-*test, and a one-way analysis of variance followed by Dunnett’s *t*-test were used to assess significant differences between groups. The *p* values < 0.05 were considered statistically significant.

## Additional Information

**How to cite this article**: Wang, X. *et al*. Sulforaphane improves chemotherapy efficacy by targeting cancer stem cells-like properties via the miR-124/IL-6R/STAT3 axis. *Sci. Rep.* **6**, 36796; doi: 10.1038/srep36796 (2016).

**Publisher’s note:** Springer Nature remains neutral with regard to jurisdictional claims in published maps and institutional affiliations.

## Supplementary Material

Supplementary Information

## Figures and Tables

**Table 1:** Sequences of the primers used for real time RT-PCR.

IL6	Forward	5′-AGTAGTGAGGAACAAGCCAGA-3′
	Reverse	5′-TACATTTGCCGAAGAGCC-3′
IL6R	Forward	5′-TCACTGTGTCATCCACGACG-3′′
	Reverse	5′-AGCCAGCTATCTGGGGAAGA-3′
STAT3	Forward	5′-CTTTGAGACCGAGGTGTATCACC-3′
	Reverse	5′-GGTCAGCATGTTGTACCACAGG-3′
CD44	Forward	5′-TGAGCATCGGATTTGAGAC-3′
	Reverse	5′-CATACTGGGAGGTGTTGGA-3′
EpCAM	Forward	5′-CTGCCAAATGTTTGGTGATG-3′
	Reverse	5′-ACGCGTTGTGATCTCCTTCT-3′
GAPDH	Forward	5′-GACCTGACCTGCCGTCTA-3′
	Reverse	5′-GGAGTGGGTGTCGCTGT-3′
