# Supplementary material for: Sulforaphane improves chemotherapy efficacy by targeting cancer stem cell-like properties via the miR-124/IL-6R/STAT3 axis
Source: Sci Rep. 2016 Nov 8;6:36796. doi: 10.1038/srep36796 (PMC5099878; doi:10.1038/srep36796)
Supplement: Supplementary Information [file srep36796-s1.pdf]

SUPPLEMENTARY DATA

Title:

Sulforaphane improves chemotherapy efficacy by targeting cancer stem cell-like properties via the miR-124/IL-6R/STAT3 axis

Authors:

Xingxing Wang [1, *], Yuan Li [1, *], Yi Dai [2, *], Qinqiang Liu [2, *], Shilong Ning [1], Jiao Liu [1], Zhaoxia Shen [1], Dongmei Zhu [1], Fei Jiang [1], Jianping Zhang [2], and Zhong Li [1, ✉]

Authors' affiliations:

1. Key Laboratory of Modern Toxicology, Ministry of Education, Department of Nutrition and Food Hygiene, School of Public Health, Nanjing Medical University, Nanjing, 211166, China.
2. Department of surgery, the second affiliated hospital, Nanjing medical university, Nanjing, 211166, China.

Authors' contributions *:

Xingxing Wang, Yuan Li, Yi Dai, and Qinqiang Liu, contributed equally to this work.

Corresponding author informations [✉]:

Dr. Zhong Li, Department of Nutrition and Food Hygiene, The Key Laboratory of Modern Toxicology, Ministry of Education, School of Public Health, Nanjing Medical University, Nanjing 211166, China. Telephone: +86-25-8686-8451. Fax: +86-25-8652-7613. E-mail: lz-ny@njmu.edu.cn.

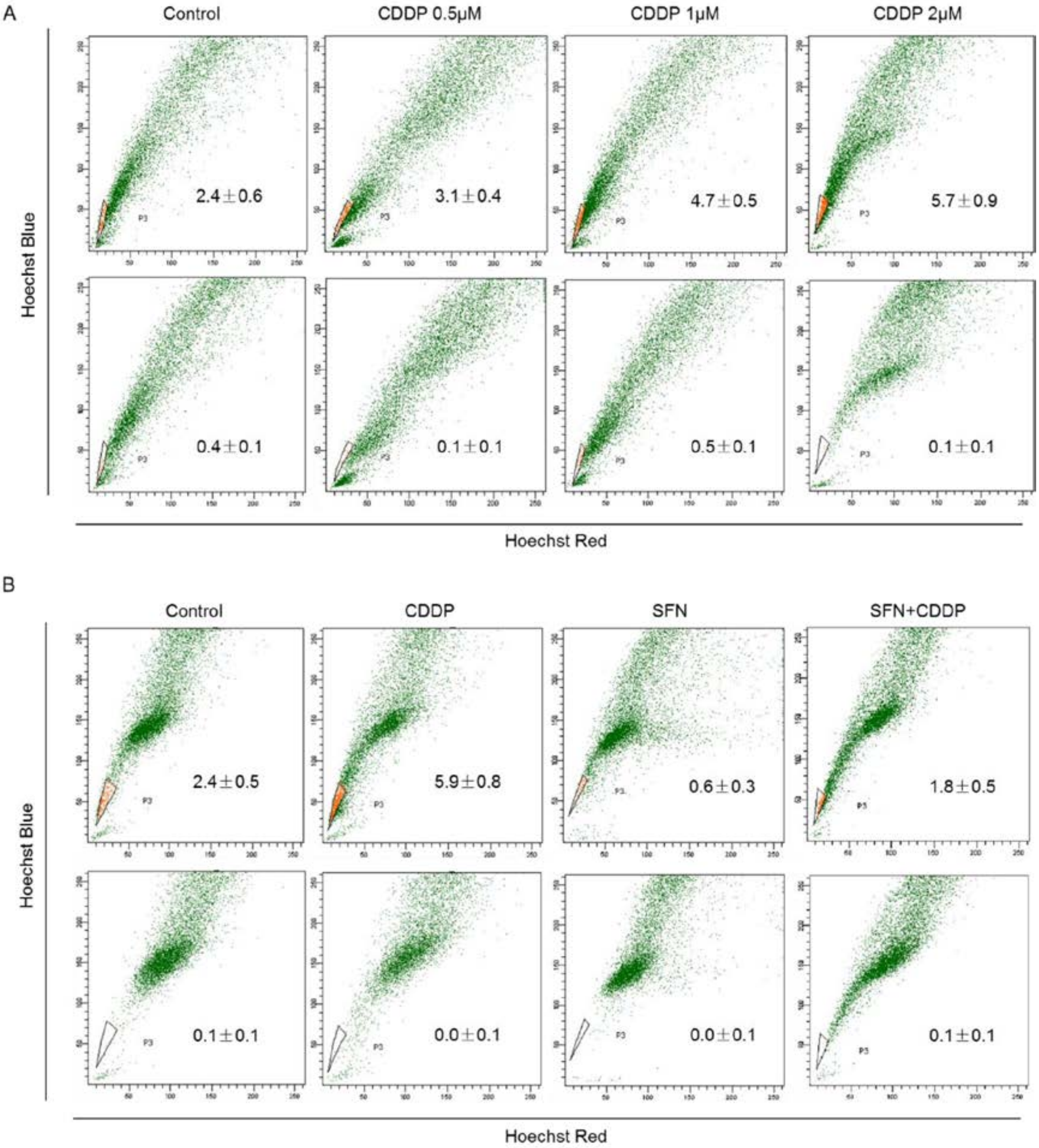
A
Control
CDDP 0.5μM
CDDP 1μM
CDDP 2μM
2.4±0.6
3.1±0.4
4.7±0.5
5.7±0.9
0.4±0.1
0.1±0.1
0.5±0.1
0.1±0.1
Hoechst Blue
Hoechst Red
B
Control
CDDP
SFN
SFN+CDDP
2.4±0.5
5.9±0.8
0.6±0.3
1.8±0.5
0.1±0.1
0.0±0.1
0.0±0.1
0.1±0.1
Hoechst Blue
Hoechst Red

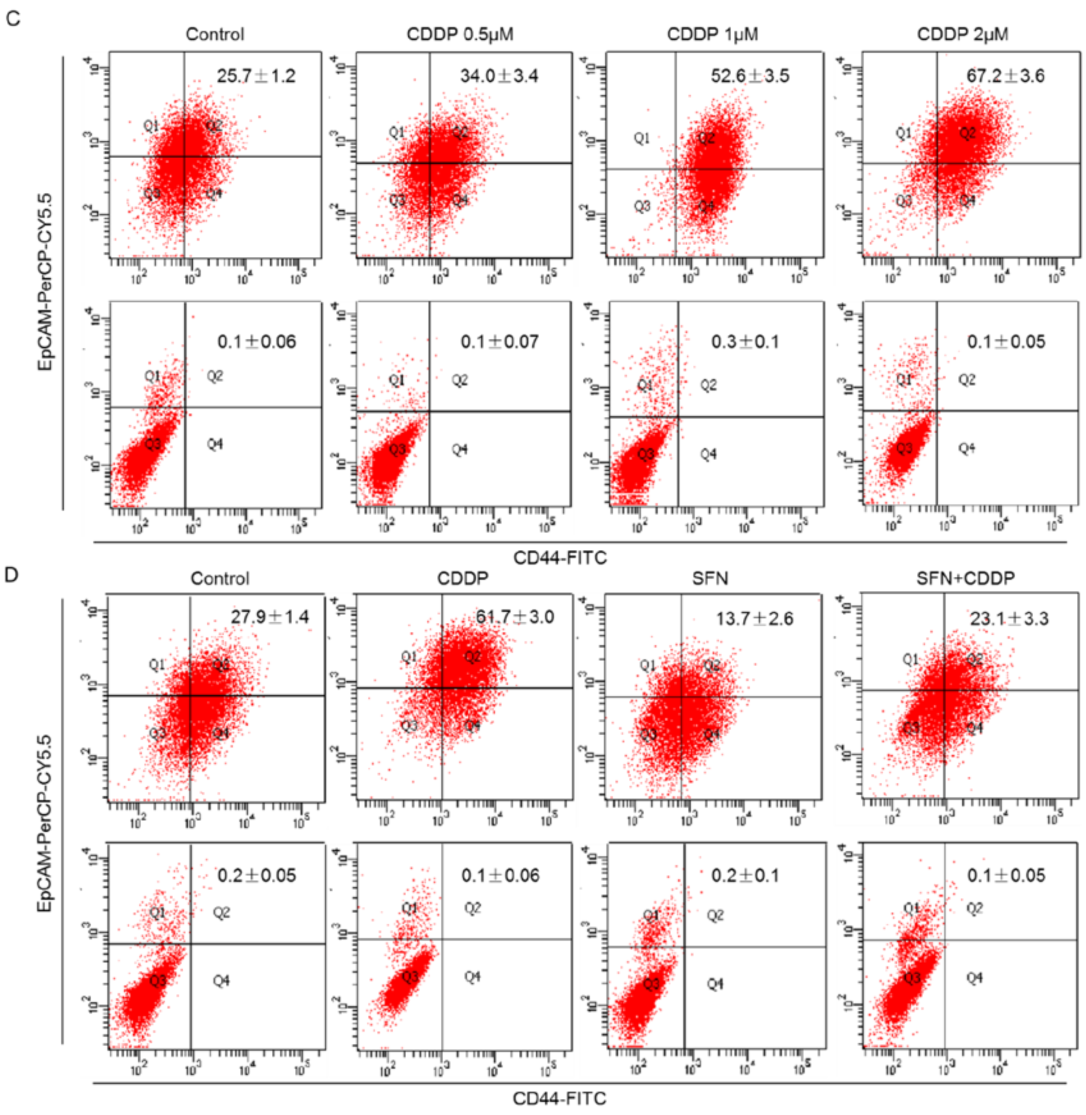

Fig. S1. The effects of SFN and CDDP on the CSC-like properties in MGC803. **(A, C)** MGC803 cells were treated with 0, 0.5, 1, or 2 μM of CDDP for 72 h. **(B, D)** MGC803 cells were treated with 2 μM of CDDP, 10 μM of SFN, or their combination for 72 h. **(A, B)** Flow cytometry analyses of the percentage of SP and **(C, D)** $CD44^+/EpCAM^+$ cells in MGC803 cell populations (mean ± SD, n = 3).

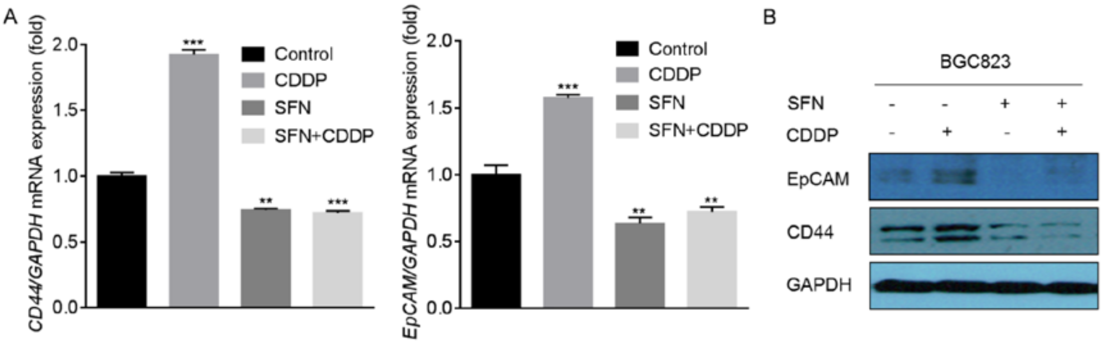

Fig. S2. SFN represses the CDDP-induced CSC surface markers in BGC823.

(A, B) BGC823 cells were treated with 2 μM of CDDP, 10 μM of SFN, or their combination for 72 h. **(A)** qRT-PCR analyses of *CD44* and *EpCAM* (mean ±SD, n=3). **$p < 0.01$ and ***$p < 0.001$compared with medium control BGC823 cells. **(B)** Western blot analysis of CD44 and EpCAM.

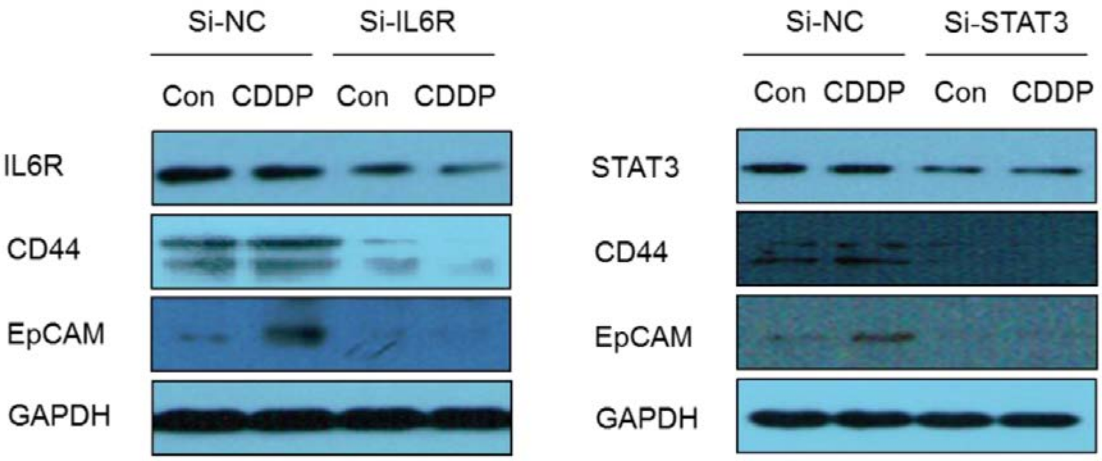

Fig. S3. IL-6/STAT3 signaling is responsible for CDDP-induced CSC surface markers in BGC823.

BGC823 cells were transfected with con-siRNA, IL6R-siRNA, or STAT3-siRNA for 12 h and then 2 μM of CDDP was added for 72 h. Western blot analysis of related proteins.

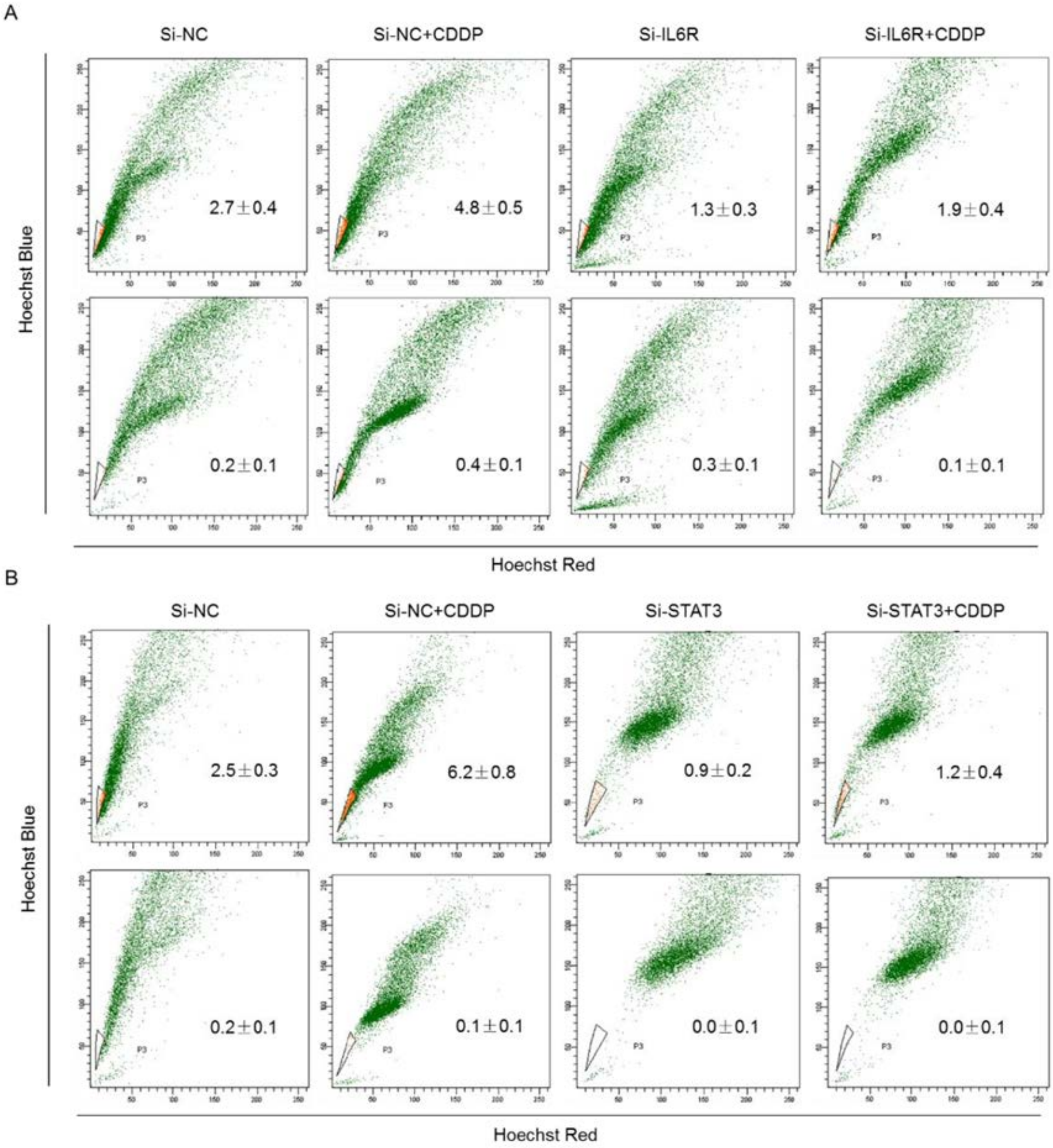
A
Si-NC
Si-NC+CDDP
Si-IL6R
Si-IL6R+CDDP
2.7±0.4
4.8±0.5
1.3±0.3
1.9±0.4
0.2±0.1
0.4±0.1
0.3±0.1
0.1±0.1
Hoechst Blue
Hoechst Red
B
Si-NC
Si-NC+CDDP
Si-STAT3
Si-STAT3+CDDP
2.5±0.3
6.2±0.8
0.9±0.2
1.2±0.4
0.2±0.1
0.1±0.1
0.0±0.1
0.0±0.1
Hoechst Blue
Hoechst Red

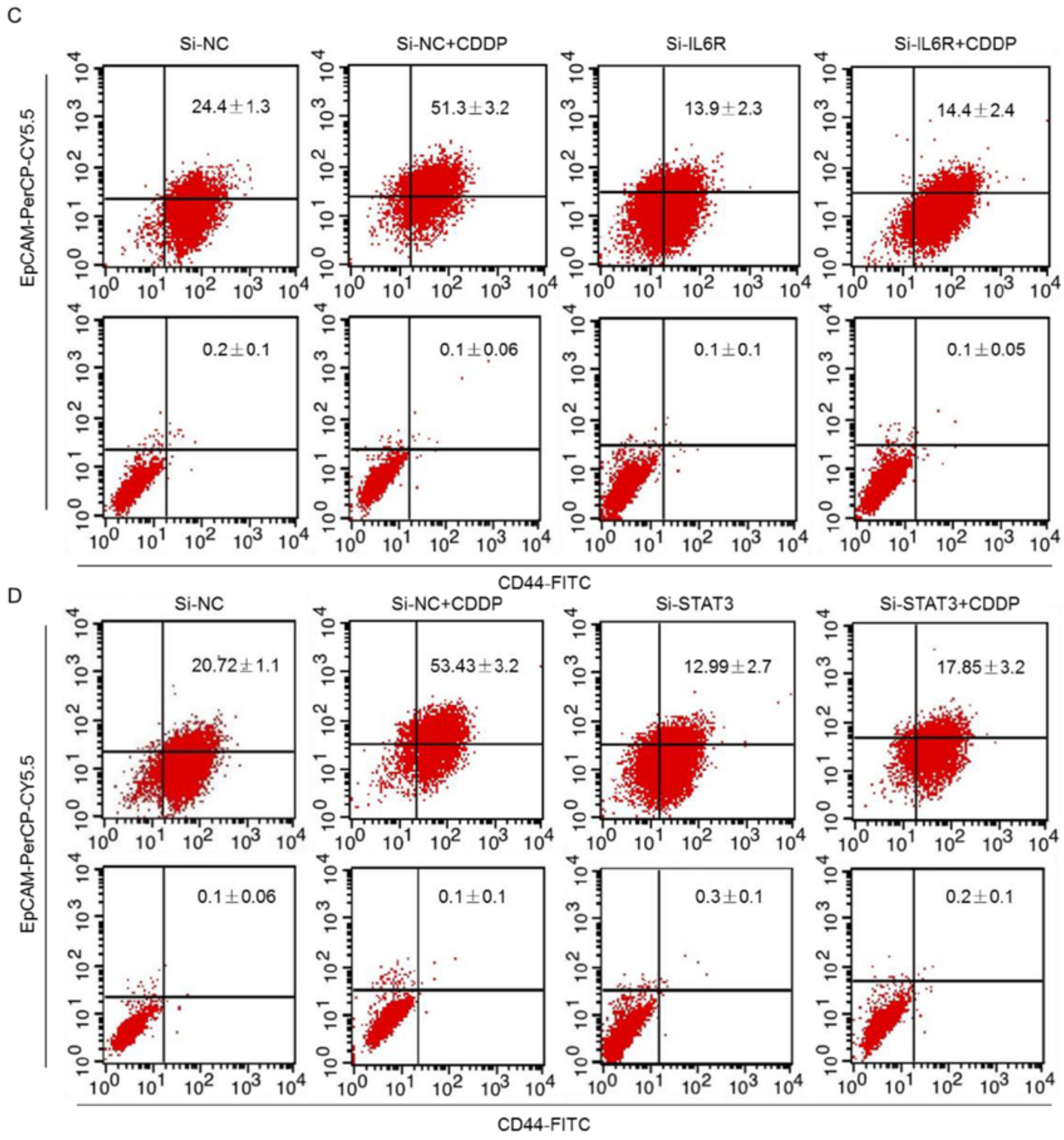

Fig. S4. IL-6/STAT3 signaling is responsible for CDDP-induced gastric CSC-like properties in MGC803.

(A-D) MGC803 cells were transfected with con-siRNA, IL6R-siRNA, or STAT3-siRNA for 12 h and then 2 μM of CDDP was added for 72 h. **(A, B)** FACS analyses of the percentage of SP and **(C, D)** $CD44^+/EpCAM^+$ cells in MGC803cell populations (mean ± SD, n = 3).

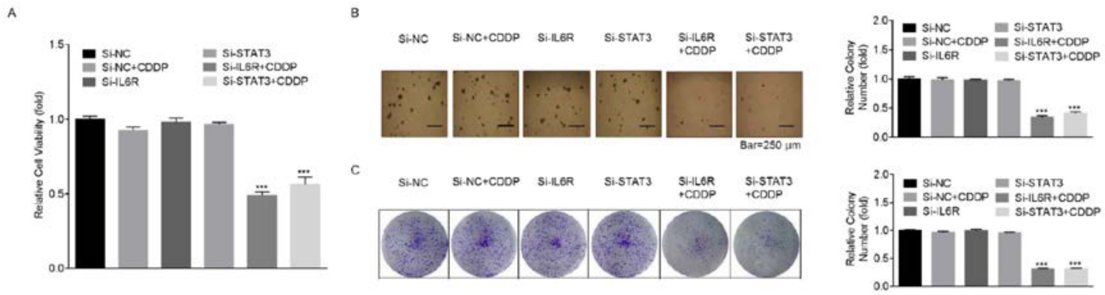

Fig. S5. Blocking IL-6/STAT3 signaling enhances the chemotherapy efficacy in BGC823.

(A-C) BGC823 cells were transfected with con-siRNA, IL6R-siRNA, or STAT3-siRNA for 12 h and then 2 μM of CDDP was added for 72 h. **(A)** Cell viability was evaluated by the CCK-8 assay. **(B, left)** Soft agar colonies under a microscope after 2 weeks of cultivation. **(B, right)** Relative colony numbers in soft agar (mean ± SD, n=5). **(C, left)** Plate colonies after 2 weeks. **(C, right)** Relative colony numbers in plates (mean ±SD, n=3). ****p* < 0.001compared with MGC803 cells transfected by con-siRNA.

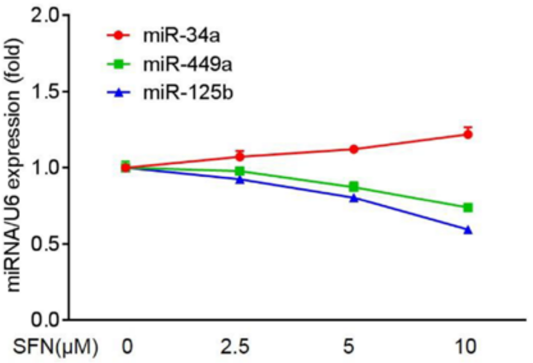

Fig. S6. The effects of SFN on miRNAs.

MGC803 cells were treated with 0, 2.5, 5, or 10 μM of SFN for 72 h. The qRT-PCR analyses of related miRNAs (mean ± SD, n=3).

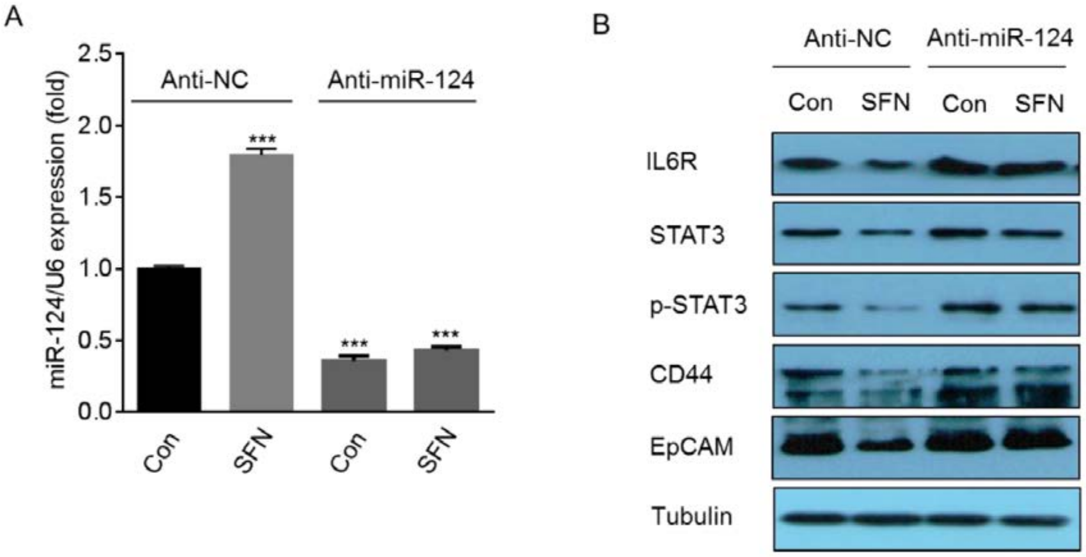

Fig. S7. SFN suppressed IL-6R, STAT3 and the CDDP-induced CSC surface markers via miR-124 in BGC823.

(A, B) BGC823 cells were transfected with anti-con or anti-miR-124 for 12 h and treated with 0 or10 μM of SFN for 72 h. **(A)** qRT-PCR analyses of miR-124 (mean ± SD, n=3). ****p* < 0.001 compared with BGC823 cells transfected with anti-con. **(B)** Western blot analysis of related proteins.

A

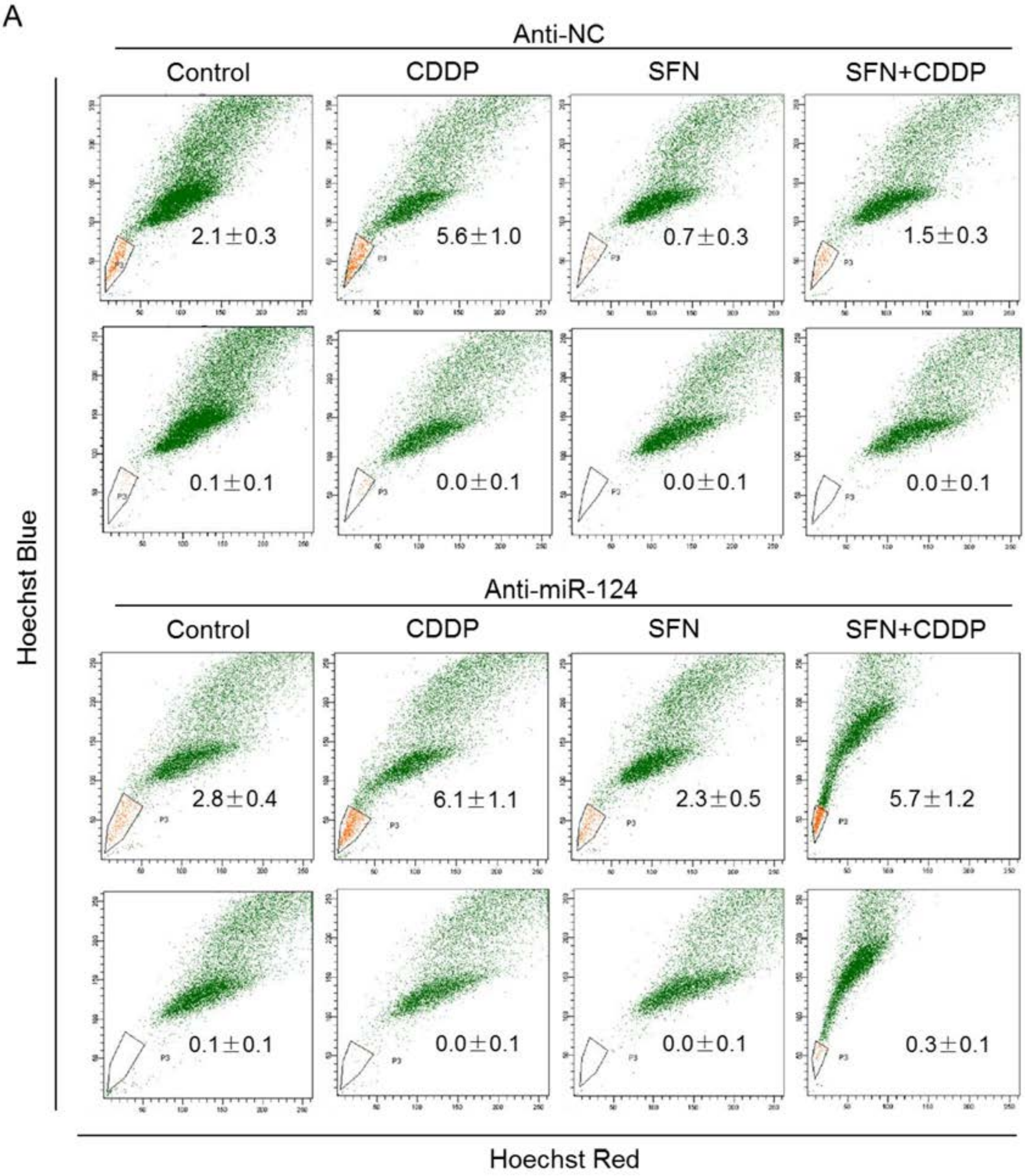
Anti-NC
Control
CDDP
SFN
SFN+CDDP
2.1±0.3
5.6±1.0
0.7±0.3
1.5±0.3
0.1±0.1
0.0±0.1
0.0±0.1
0.0±0.1
Anti-miR-124
Control
CDDP
SFN
SFN+CDDP
2.8±0.4
6.1±1.1
2.3±0.5
5.7±1.2
0.1±0.1
0.0±0.1
0.0±0.1
0.3±0.1
Hoechst Blue
Hoechst Red

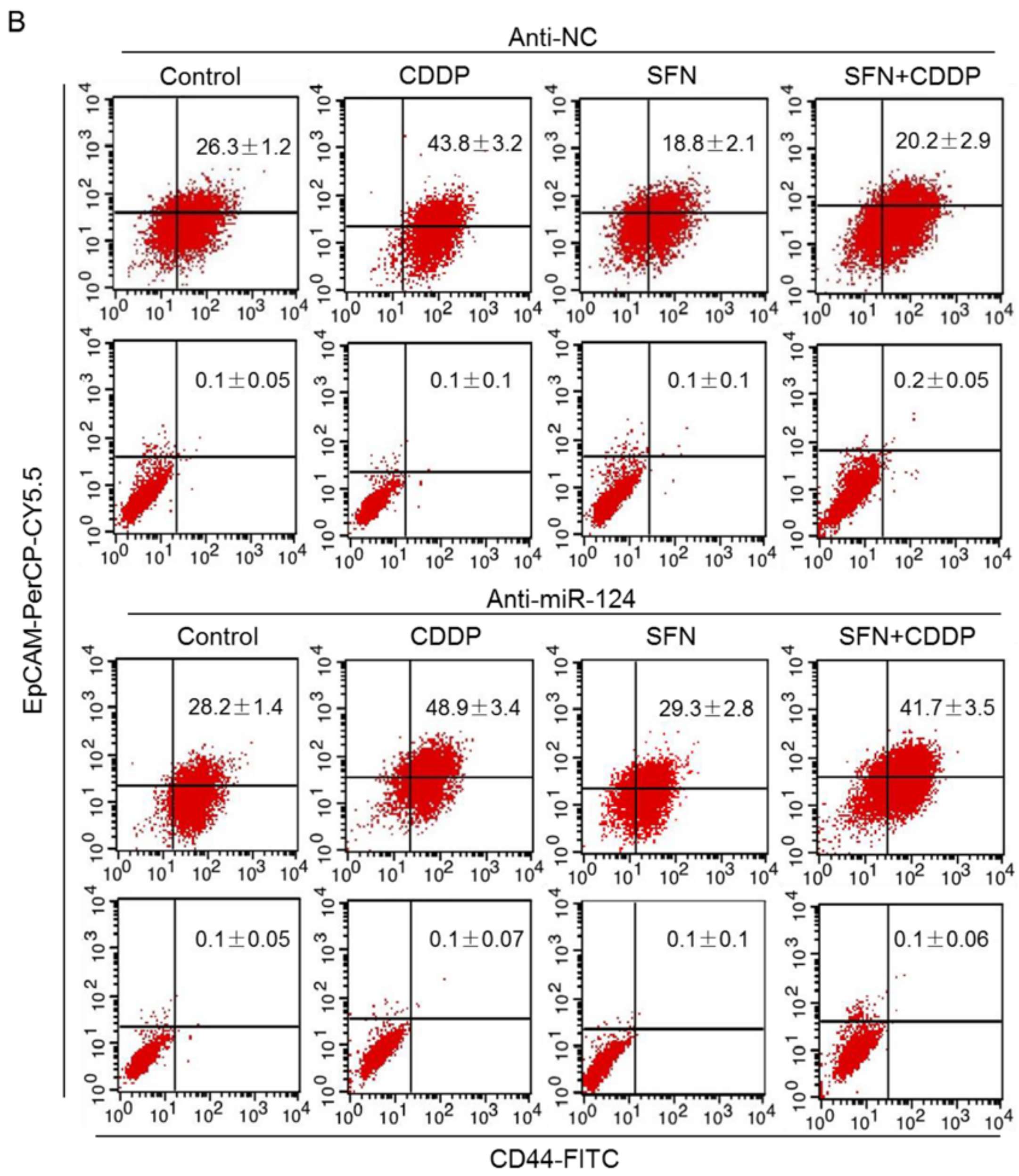

Fig. S8. SFN decreases the CDDP-induced CSC-like properties via miR-124.

(A, B) MGC803 cells were transfected with anti-con or anti-miR-124 for 12 h and treated with 2 μM of CDDP, 10 μM of SFN, or their combination for 72 h. FACS analyses of the percentage of SP **(A)** and $CD44^+/EpCAM^+$ **(B)** cells in MGC803 cell populations (mean ± SD, n = 3).
